# Data on TREM-1 activation destabilizing carotid plaques

**DOI:** 10.1016/j.dib.2016.05.047

**Published:** 2016-05-27

**Authors:** Velidi H. Rao, Vikrant Rai, Samantha Stoupa, Saravanan Subramanian, Devendra K. Agrawal

**Affiliations:** Department of Clinical and Translational Science, Creighton University School of Medicine, Omaha, NE 68178, United States

**Keywords:** Triggering receptor expressed on myeloid cells-1, Carotid plaques, Atherosclerosis, Matrix metalloproteinases, Macrophages, Vulnerable plaque, Unstable plaque, Vascular smooth muscle cells

## Abstract

The data described herein are related to the article entitled “Tumor necrosis factor-α regulates triggering receptor expressed on myeloid cells-1-dependent matrix metalloproteinases in the carotid plaques of symptomatic patients with carotid stenosis” (Rao et al., 2016) [1]. Additional data are provided on the dose–response effect of TNF-α, TREM-1 antibody and recombinant rTREM-1/Fc fusion chimera (TREM-1/FC) on the expression of MMP-1 and MMP-9 in vascular smooth muscle cells (VSMCs) isolated from human carotid endarterectomy tissues. Data are also presented on the distribution of CD86+ M1- and CD206+ M2-macrophages and their co-localization with TREM-1 in symptomatic carotid plaques as visualized by dual immunofluorescence. The interpretation of this data and further extensive insights can be found in Rao et al. (2016) [1].

Specifications Table

**Table:** 

Subject area	Health sciences
More specific subject area	Atherosclerosis
Type of data	Figures
How data was acquired	Fluorescent microscope (Olympus BX51), Real-time PCR system model CFX96 (BioRad Laboratories, Herculus, CA). Image analysis: ImageJ prosoftware
Data format	Analyzed
Experimental factors	VSMCs and carotid endarterectomy tissues
Experimental features	The isolated VSMCs were treated with TNF-α with or without TREM-1 antibody and recombinant rTREM-1/Fc fusion chimera (TREM-1/FC). qPCR was used for mRNA expression and protein expression by immunofluorescence studies.
Data source location	Department of Clinical and Translational Science, Creighton University School of Medicine, Omaha, NE 68178
Data accessibility	Data within the article

**Value of the data**

• The data provide the information on the dose–response effect of TNF-α on the expression of TREM-1, MMP-1 and MMP-9. This information can be used by the researchers to select a dose for their experiments.
• Data are also presented to provide information on the individual dose–response effect of both TREM-1 antibody (4–20 ug/ml) and TREM-1 decoy receptor rTREM-1/Fc (0.2–1.6 μg/ml) on the expression of TREM-1, MMP-1 and MMP-9 in TNF-α -treated VSMCs. Such information is valuable to the scientific community/researchers to select a dose for their experiments.
• Data are also presented on the relative distribution of CD86+ M1- and CD206+ M2-macrophages and their co-localization with TREM-1 as visualized by dual immunofluorescence.

## 1 Data

**Fig. 1:**
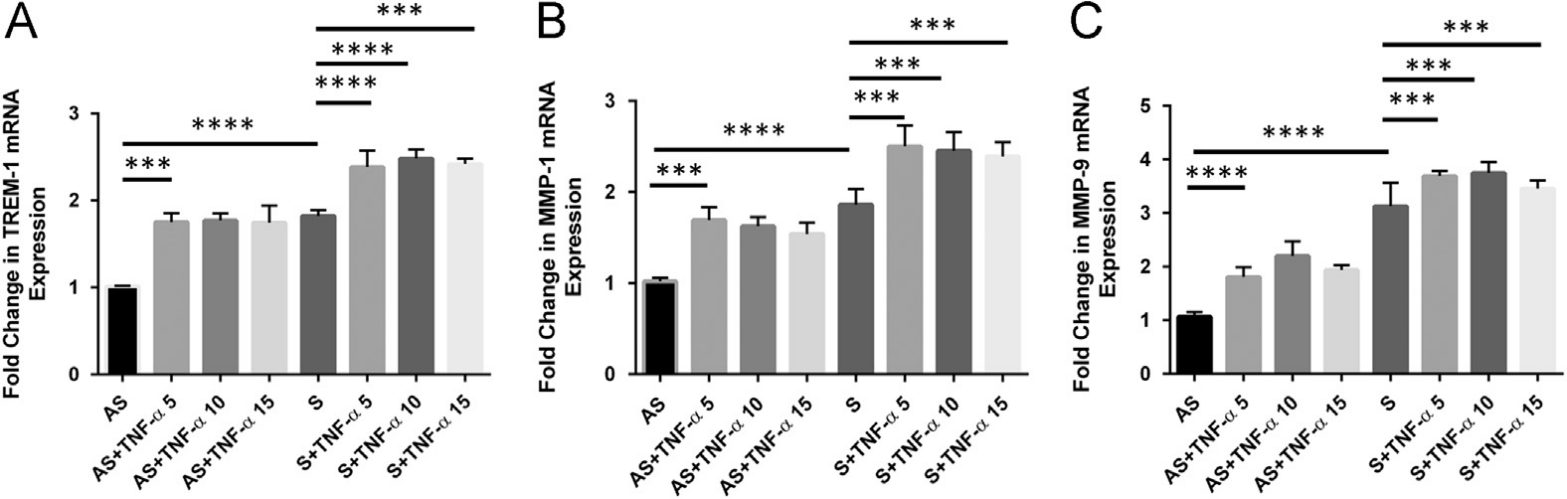
Effect of various concentrations of TNF-α (5, 10 and 15 ng/ml) treatment for 24 h on the expression of TREM-1, MMP-1 and MMP-9 in VSMCs from AS and S carotid plaques. The RNA samples isolated from the VSMCs were subjected to qPCR. Panel A, TREM-1; Panel B, MMP-1; and Panel C, MMP-9. Data are presented as mean±SD (*N*=3). Relative expression was normalized to GAPDH. *p<0.05, **p<0.01, ***p<0.001, ****p< 0.0001.

**Fig. 2:**
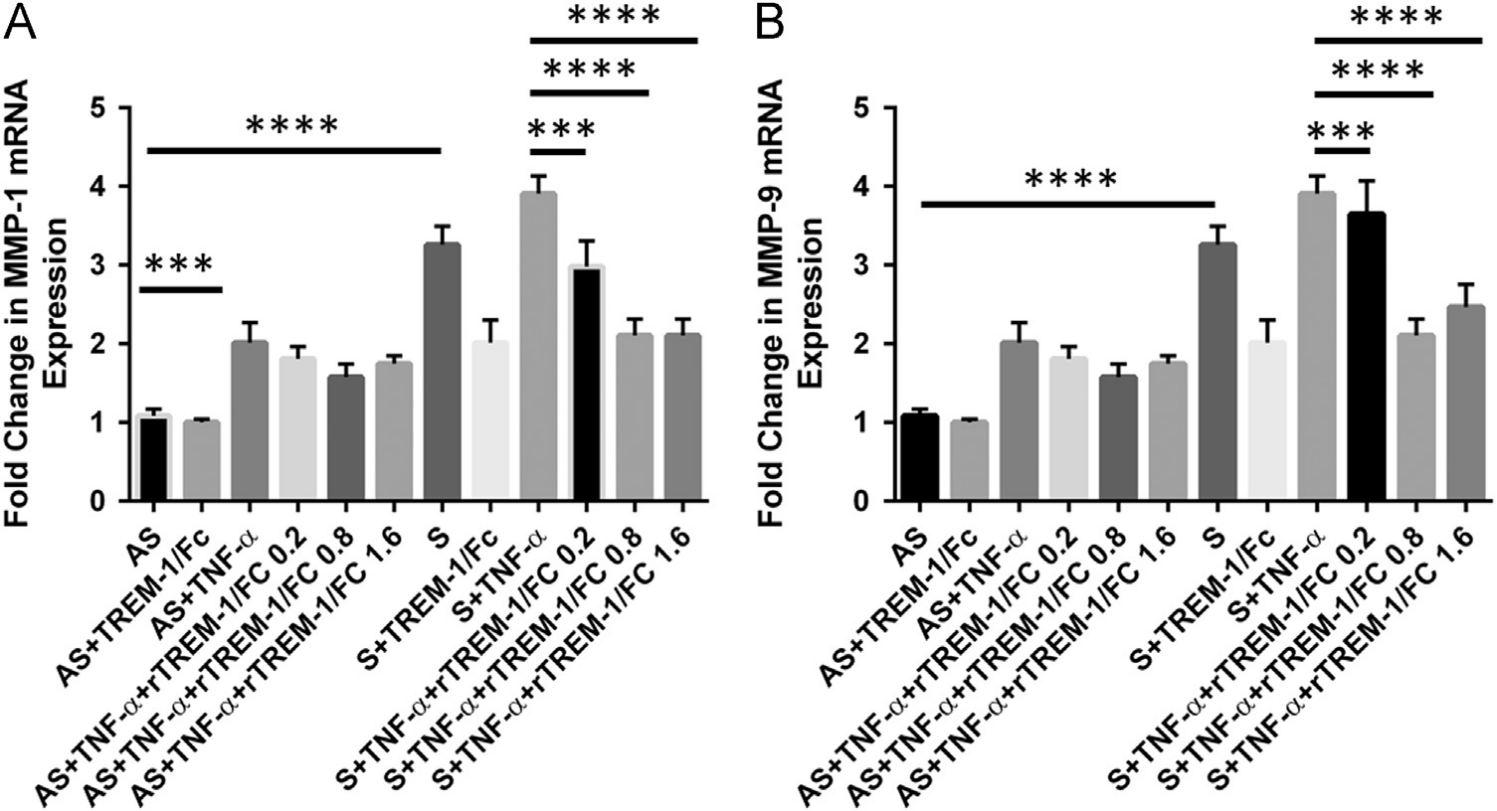
Dose-dependent effect of recombinant rTREM-1/Fc fusion chimera (TREM-1/FC) (0.2, 0.8 and 1.6 µg/ml) on the expression of MMP-1 (Panel A) and MMP-9 (Panel B) in VSMCs isolated from asymptomatic (AS) and symptomatic (S) carotid plaques and treated with TNF-α (10 ng/ml). Data are presented as mean±SD (*N*=3). Relative expression was normalized to GAPDH. *p<0.05, **p<0.01, ***p<0.001, ****p< 0.0001.

**Fig. 3:**
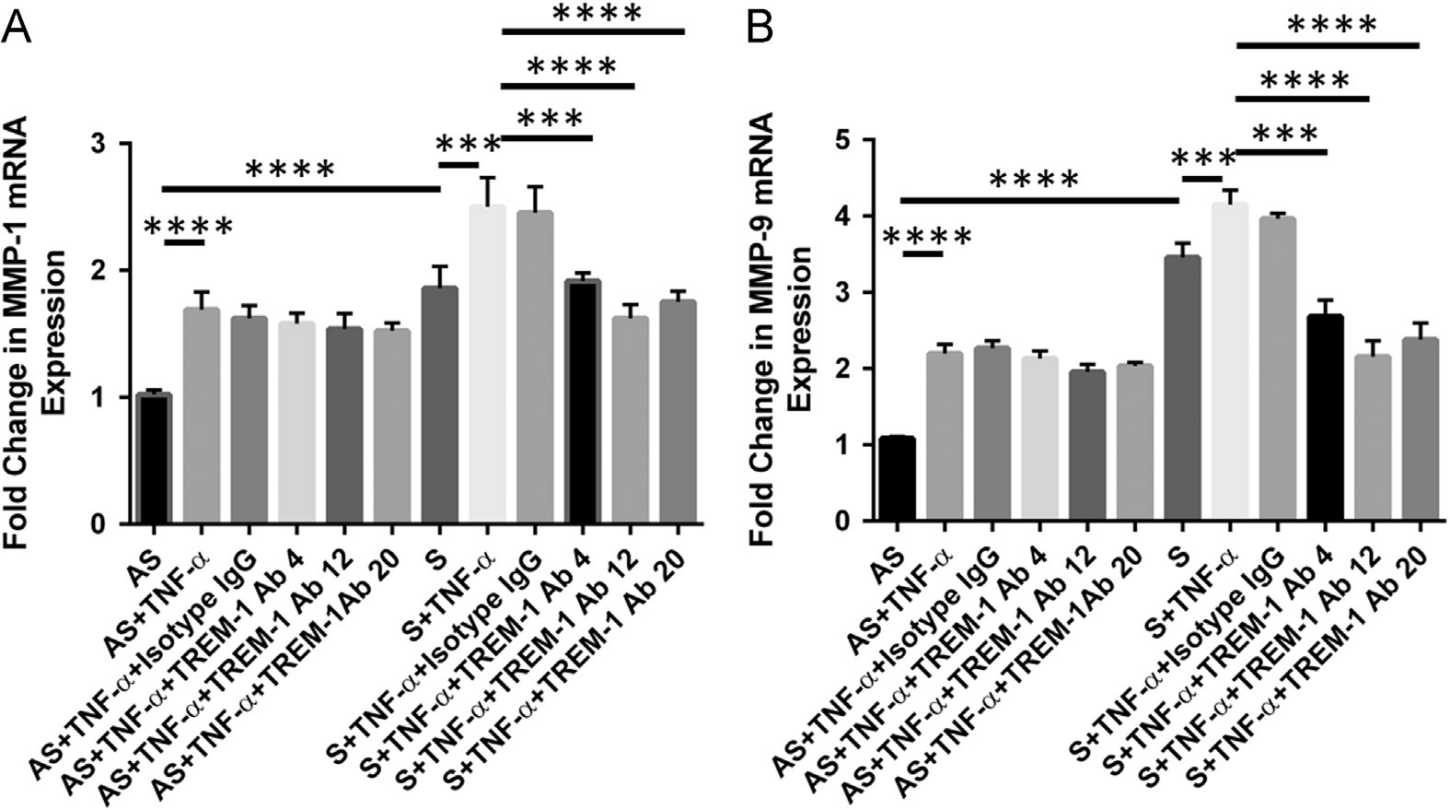
Dose-dependent effect of TREM-1 antibody (4, 12 and 20 µg/ml) on the expression of MMP-1 (Panel A) and MMP-9 (Panel B) in VSMCs isolated from asymptomatic (AS) and symptomatic (S) carotid plaques and treated with TNF-α (10 ng/ml). Data are presented as mean±SD (*N*=3). Relative expression was normalized to GAPDH. *p<0.05, **p<0.01, ***p<0.001, ****p< 0.0001.

**Fig. 4:**
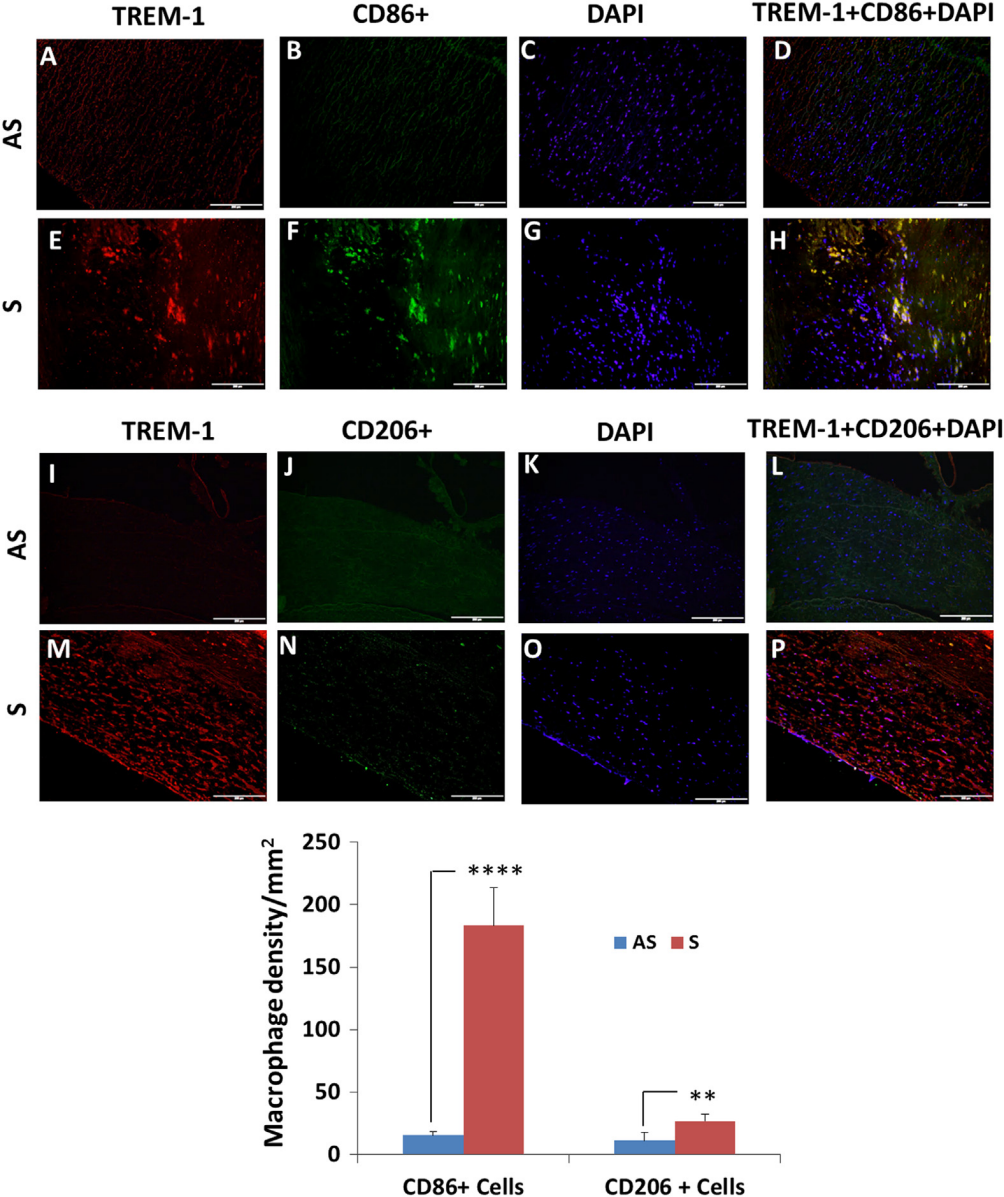
Immunofluorescence staining of TREM-1, CD86+ M1-macrophages and CD106+ M2-macrophages in tissue sections of asymptomatic (AS) and symptomatic (S) carotid plaques. Representative images are shown for TREM-1 (red) and CD86 (green) expression as visualized by dual immunofluorescence in the tissue sections of asymptomatic (AS: Panels, A-D) and symptomatic (S: Panels, E-F). Co-localization of TREM-1 (red) and CD206 (green) is shown for AS (Panels I-L) and S (Panels M-P). Density of CD86^+^ M1- and CD206^+^ M2-macrophages was counted per mm^2^ from 5 different tissues in each experimental group and the data are shown in the bar graph (lower panel). Data are presented as mean±SD; *N*=5. *p<0.05, ***p<0.001, ****p<0.0001. Scale bar=200 µm for all images.

In Fig. 1, data show the dose–response effect of TNF-α (5, 10 and 15 ng/ml) on the expression of TREM-1, MMP-1 and MMP-9. Data are also presented on the individual dose-dependent effect of both recombinant rTREM-1/Fc fusion chimer (0.2, 0.8, and 1.6 µg/ml) and TREM-1 antibody (4, 12 and 20 µg/ml) on the expression of MMP-1 and MMP-9 in VSMCs isolated from AS and S carotid plaques treated with 10 ng/ml of TNF-α (Fig. 2, Fig. 3). Data are also presented on the expression of TREM-1 in CD68+ M1-macrophages and CD206+ M2-macrophages (Fig. 4)**.**

## 2 Experimental design, materials and methods

### 2.1 Study subjects and acquisition of carotid endarterectomy specimens

The institutional Review Board of Creighton University approved the research protocol as exempted. Surgical specimens of human atherosclerotic plaques from carotid artery were obtained anonymously from both asymptomatic (AS) and symptomatic (S) patients, who were males and females of any ethnicity, aged 50–75 years. The carotid endarterectomy specimens were categorized as symptomatic or asymptomatic based on the clinical symptoms (2,3).

### 2.2 Isolation of VSMCs and treatment protocol

VSMCs were isolated from carotid plaques by the method previously reported by us [1], [2], [3]. VSMCs at pre-confluence were incubated with different concentrations of TNF-α at 4, 10 and 20 ng/ml for 24 h or 10 ng/ml TNF-α in the presence or absence of either recombinant rTREM-1/Fc (0.2–1.6 µg/ml) or TREM-1 antibody (4–20 µg/ml).

### 2.3 Immunofluorescence staining

The thin sections (5 µm) of the carotid plaque tissues embedded in paraffin were immunostained with the antibodies and the immunofluorescence was examined and the intensity was quantified using Image-pro software, as reported previously [1], [2].

### 2.4 Real-time qPCR

The Syber Green Master Mix was used to perform RT-qPCR (BioRad CFX96). Details of the primers have been previously described [1], [2]. Relative expression was normalized to GAPDH.

### 2.5 Inhibition of TREM-1

To examine the effect of TREM-1 inhibition, VSMCs were treated with TNF-α for 24 h in the presence or absence of either recombinant TREM-1/Fc fusion chimera (0.2, 0.8 and 1.6 µg/ml) or TREM-1 antibody (4, 12 and 20 µg/ml). The mRNA expression of MMP-1 and MMP-9 was analyzed by qPCR and relative expression was normalized to GAPDH.

### 2.6 Statistical analysis

All data are reported as mean±SD. The Student׳s *t*-test and ANOVA were used to analyze statistical differences between the experimental groups. ^*^p<0.05, **p<0.01, ***p<0.001, ****p< 0.0001.
